# Thermomechanical Properties of Ramie Fiber/Degradable Epoxy Resin Composites and Their Performance on Cylinder Inner Lining

**DOI:** 10.3390/ma17194802

**Published:** 2024-09-29

**Authors:** Jingqi Geng, Jiale Lyu, Yingchun Cai

**Affiliations:** 1 Key Laboratory of Bio-Based Material Science and Technology, Ministry of Education, School of Material Science and Engineering, Northeast Forestry University, Harbin 150040, China; gengjingqi@nefu.edu.cn; 2 School of Chemical Engineering and Technology, Harbin Institute of Technology, Harbin 150001, China; jl_lyu9508@163.com

**Keywords:** ramie fiber, degradable epoxy, thermomechanical properties, gas cylinder

## Abstract

Type IV gas cylinders are widely used in the field of vehicles due to their advantages such as light weight, cleanliness, and low cost. Ramie fiber/degradable epoxy resin composites (RFRDE) provide new ideas for the material selection of Type IV gas cylinders due to their advantages of low carbon emissions, low environmental pollution, and renewable resource utilization. However, the poor interfacial bonding strength and moisture resistance between polyethylene plastics and RFRDE have limited their application areas. This study tested the mechanical properties of ramie fibers at different heat treatment temperatures, and studied the thermal mechanical properties of RFRDE through differential scanning calorimeter and curing kinetics methods. At 180 °C, the tensile strength of fiber bundles decreased by 34% compared to untreated fibers. As the highest curing temperature decreases, the tensile strength of RFRDE increases but the curing degree decreases. At the highest curing temperature of 100 °C, the tensile strength of RFRDE is 296 MPa. The effect of the corona discharge and flexible adhesive on the surface modification of polyethylene was analyzed using scanning electron microscopy. These results provide guidance for the development of natural fiber/degradable epoxy resin composite materials.

## 1. Introduction

Hydrogen fuel cell vehicles play a significant role in harnessing hydrogen energy and achieving carbon neutrality. High-pressure gas cylinders, specifically Type IV cylinders, are key to onboard hydrogen storage technology [1,2,3]. These cylinders primarily consist of a lightweight plastic liner wrapped with reinforced fibers, and are chosen for their low cost, light weight, and high durability [4,5]. Type IV high-pressure hydrogen storage cylinders currently face numerous technical challenges that necessitate research across various domains, including material design, plastic liner and mold design, failure analysis, structural design, and optimization of the fiber-wrapped layer [6,7,8]. Among these, a common issue with Type IV high-pressure cylinders is the poor interface of the plastic liner and fibers, which has become a focal point for research efforts.

During the inflation and expansion process of hydrogen cylinders, gas continuously permeates and dissolves into the plastic liner [1,9,10]. In this process, a rapid pressure drop or evacuation inside the cylinder can easily lead to a pressure differential at the plastic liner–fiber interface, causing delamination [11]. Improving the interfacial adhesion strength is beneficial for coordinating deformation during the expansion of the cylinder, reducing the deformation of the liner layer [12,13]. Dehaghani et al. [14] tested the effect of acid etching duration on the interfacial bonding performance of polyester (PE) and glass fiber reinforced plastics (GFRPs). Their results showed that increased acid etching time led to a decrease in the PE surface contact angle, thereby enhancing the interfacial bonding strength and improving resistance to crack deformation.

Ramie fiber, extracted from the ramie plant, is a natural fiber that possesses many advantages [15,16]. It is highly resistant to damage from abrasion and stretching, making it suitable for durable fabrics. Masseteau et al. [17] investigated the effect of moisture content on the properties of flax fibers and their composites, and studied the tensile properties of flax fibers and their composites with different moisture contents. The results showed that the tensile modulus of flax fibers with a high moisture content was reduced by 20.87% compared to those with a low moisture content. Alamri and Low [18] experimented with varying fiber loadings of 19, 28, 40, and 46 wt.% to produce recycled cellulose fiber (RCF)-reinforced epoxy composites. Their results demonstrated that as the fiber content increased, there were corresponding improvements in the flexural strength, flexural modulus, fracture toughness, and impact strength. The maximum mechanical properties were observed at the highest fiber loading of 46 wt.%. Additionally, chemical treatments of the fibers reduce the moisture content and remove impurities that hinder adhesion between the fibers and the matrix, resulting in enhanced mechanical strength [19]. Plasma treatment of the fibers further contributes to improving these mechanical properties [20]. However, the hydroxyl and carboxyl groups on the surface of ramie fibers easily form hydrogen bonds with water molecules, and the rich porous structure inside the fibers is conducive to water adsorption, resulting in a weakened interfacial strength between ramie fibers and the resin matrix [21,22].

This article first proposes the use of fully biodegradable composite materials for the preparation of pressure vessels using a winding process, and systematically studies the resin thermal curing process as well as the effect of hemp fiber heat treatment on resin-based composite materials. The chemical inertness on the surface of PE makes it difficult to adhere, which affects the interface bonding between it and ramie fiber composite materials; this in turn makes it difficult for the fiber layer of the pressure vessels to play a load-bearing role. Therefore, three methods were adopted to increase the interface strength between the PE lining and ramie fibers, namely ca orona treatment, an adhesive treatment, and a corona + adhesive treatment. When the fiber winding tension is 1.5 kg, the maximum burst pressure obtained from the last four types of gas cylinders is 14.1 MPa, which is similar to the design burst pressure of 15 MPa. Analysis of the gas cylinder after blasting reveals that the damage mainly manifests itself as a fiber fracture and interface delamination.

## 2. Materials and Methods

### 2.1. Materials

**Figure 1:**
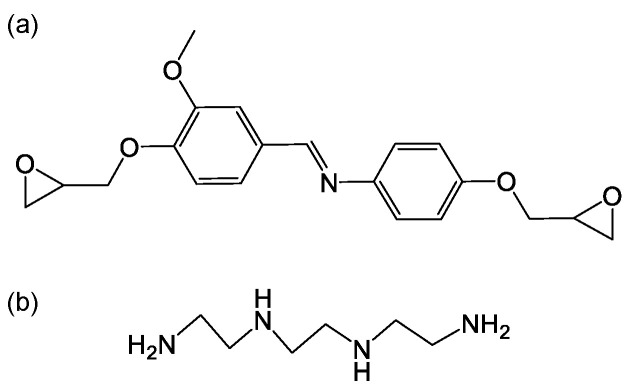
Chemical structure of (**a**) epoxy resin and (**b**) hardener.

Degradable epoxy resin was provided by Aida Suo Wuhu Co., Ltd., Wuhu, China. The chemical structure of the epoxy resin and hardener (0.98 gm/cm^3^, supplied by Singhal Chemical Corporation, Uttar Pradesh, India) is shown in Figure 1. Ramie fiber (*Boehmeria nivea* (L.) Gaudich) was obtained from Qichun County Dongshen Textile Raw Materials Co., Ltd., Huanggang, China.

### 2.2. Testing Instruments and Conditions

#### 2.2.1. Heat Treatment of Ramie Fiber

A group of raw ramie (10 g) was evenly wrapped on a self-made mesh mold to ensure that each raw ramie was evenly heated, which was conducted on a HB-Q3000 heat-setting machine (Ludhiana Dyeing Machinery Works, Ludhiana, India). The measured temperature and time are 100 °C/1 h, 125 °C/1 h, 150 °C/1 h, and 180 °C/1 h, respectively.

#### 2.2.2. Differential Scanning Calorimeter (DSC) Measurements

The DSC measurements were conducted on Mettler Toledo DSC-1 with different heating rates of 5 °C/min, 10 °C/min, 15 °C/min, and 20 °C/min within the temperature range of 25–300 °C.

#### 2.2.3. Curing Kinetics of RFRDE

The degree of conversion or extent of the curing reaction and the reaction rate as a function of time can be calculated from the DSC data by the following equations [23]:

*α* = *H*_t_/∆*H*_0_ (1)

d*α*/d*t* = [*A* exp(−*E*_a_/*RT*)](1 − *α*)*n* (2)

where *α* denotes the degree of conversion, *H*_t_ is the reaction enthalpy within time *t*, and ∆*H*_0_ is the total enthalpy of the curing reaction. dα/d*t* is the rate of reaction and *A* is the pre-exponential factor; *R* is the ideal gas constant, *E*_a_ is the apparent activation energy of the curing reaction, and *n* is the reaction order.

#### 2.2.4. Thermal Properties

The thermal stability of the synthesized composites was evaluated using thermogravimetric analysis (TGA) with a Pyris 1 TGA apparatus from Perkin Elmer, Waltham, MA, USA. About 5 mg of the dried sample, placed in a sealed aluminum pan, was heated from room temperature to 550 °C at a rate of 10 °C/min in a nitrogen atmosphere. The degradation onset temperature and the remaining mass were derived from the TGA curve.

#### 2.2.5. Mechanical Properties

The mechanical properties of composite materials are tested on the Instron Model testing machine. The length of bundled yarn tensile specimen is 150 mm, with the testing standard ASTM D4018-99. The tensile specimen size of composite materials is 250 mm × 15 mm, and the testing standard is based on ASTM D3039 [24]. Adhesion strength testing was conducted using polyethylene-ramie fiber composite material (PE/RFRDE) samples, with a sample size of 100 mm × 25 mm, according to the testing standard ISO 4587:2003 [25].

#### 2.2.6. PE/RFRDE Adhesive Strength Test

The size of the PE/RFRDE plate is 100 mm × 25.5 mm × 2 mm. Loctite EAE-120HP epoxy resin adhesive was chosen as the adhesive. According to the ASTM D5868-01 standard, three types of PE/RFRDE specimens were subjected to tensile testing on the Instron5582 hydraulic suit universal material testing machine, with corona treatment (1#), adhesive treatment (2#), and corona + adhesive treatment (3#). The detailed steps for the corona treatment are: Various electrode configurations for the corona plasma generator, including multi-point and flat electrodes, were utilized. The electrodes were connected to a 30 kV power supply, operating under ambient gas conditions, atmospheric pressure, and constant room temperature. In this experiment, 25 tipped bolts served as the multi-point electrodes, connected to a 2 MΩ resistor and a 20 μF capacitor. The samples were placed between flat electrodes, which were connected to a 20 kΩ resistor. The multi-point electrodes were positioned as positive electrodes, perpendicular to the flat electrodes. A high-voltage multi-tester was used to measure the A.C. input and D.C. output voltages. The detailed steps for adhesive treatment are: A group of raw ramie (10 g) was evenly wrapped on a self-made mesh mold to ensure that each raw ramie was evenly heated. Then, a small droplet of the epoxy resin and hardener mixture was carefully applied to the fiber using a needle point. The resin micro-droplet was then cured at a temperature sequence of 60 °C for 2 h followed by 100 °C for 5 h. During these processes, the tensile rate was 5 mm/min, and the temperature was room temperature. The maximum load at failure was recorded, and the bonding strength of the PE/RFRDE adhesive specimens was calculated.

### 2.3. Scanning Electron Microscope

The surfaces of as-prepared materials were investigated by using scanning electron microscopy (SEM, SU8010, Hitachi, Tokyo, Japan) with an acceleration voltage of 10 kV. As-prepared composites were stuck into the substrate with carbon glue. To enhance conductivity prior to examination, the surface of the specimen was covered with a thin layer of gold-palladium by sputter-coating in a vacuum chamber.

### 2.4. PE-RFRDE Gas Cylinder Winding

The winding of a cylinder in a fiber layer is carried out by continuously winding two circumferential winding layers and two spiral winding layers. At the dome, there is no fiber layer wrapped in a circumferential direction. According to the radius of the rod hole and the radius of the cylinder, the winding angle of the composite material container is 18°. According to research, the combination of spiral winding and circumferential winding can enhance the load-bearing capacity and prolong the fatigue life of gas cylinders.

## 3. Results

### 3.1. Mechanical Properties of Ramie Fibers with Heat Treatment

**Figure 2:**
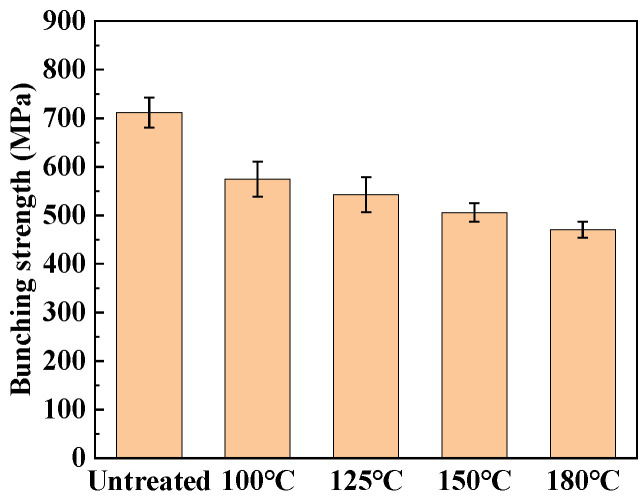
Effect of different heat treatment temperatures on the bunching strength of ramie fiber. Error bars represent the standard deviation of five representative measurements.

First, the relationship between heat treatment temperatures and bunching strength was thoroughly investigated. Figure 2 shows different heat treatment temperatures on the bunching strength of ramie fiber. It can be seen that the tensile strength of ramie fiber bundles decreases with the increase in the heat treatment temperature. The decline in percentage of bunching tensile strength at different temperatures is shown in Table 1. When the heat treatment temperature reaches 180 °C, the tensile strength of the bundles decreases by 34% compared to the untreated samples. A small amount of water on the surface of ramie fibers can improve the bonding strength between fibers. Due to the formation of a water film on the fiber surface, the friction between fibers is increased. Although the strength of ramie fiber is improved by a small amount of water absorption, the interface performance and resin performance of the composite material will decline. However, the stability and strength of the entire fiber structure are increased, which is comparable with composites based on synthetic fibers [26].

**Figure 3:**
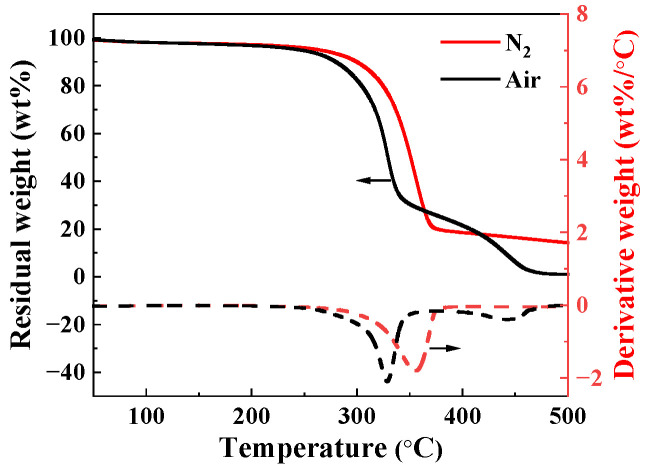
Residual weight and derivative weight curves against temperature, heat treatment under nitrogen and air atmosphere.

Then, the residual weight of ramie fibers under two different atmospheres (air and nitrogen) was characterized and is shown in Figure 3. Both curves exhibit a stepped shape, and the residual weight percentage of fibers decreases exponentially at around 300 °C. When the temperature rises to 350 °C, the residual weight percentage of fibers in N_2_ decreases slowly, while the weight of fibers in air decreases rapidly. When the temperature rises to 500 °C, the residual weight percentage of fibers in N_2_ is about 17%. From the derivative weight curves, it can also be seen that the thermal weight loss effect of ramie fibers is significant in the temperature range of 300–400 °C. After high-temperature and surface treatment, the water film on the fiber surface is damaged, leaving the fiber surface unprotected, which leads to a decrease in mechanical properties. Moreover, if the temperature is too high, the thermal motion of molecules inside the fiber will increase. This affects the interaction between hydrogen bonds and other structures, making the fiber softer and more prone to deformation. However, the interface of ramie fibers is more easily damaged, leading to a decrease in fiber strength.

### 3.2. Curing Kinetics Model of Degradable Epoxy Resin

**Figure 4:**
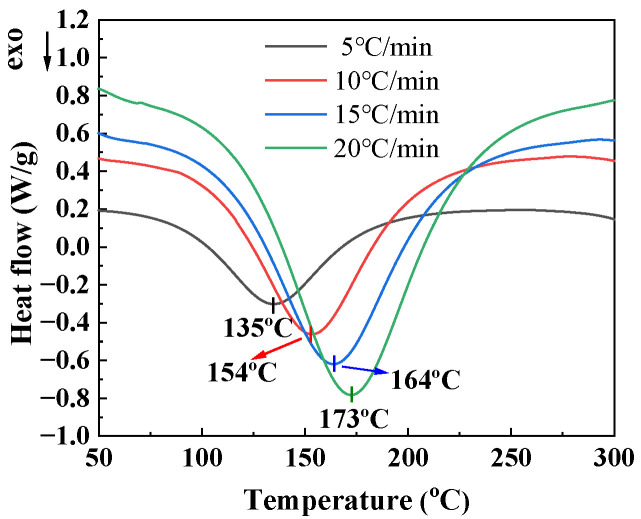
DSC curves of the degradable epoxy resin at different heating rates.

The DSC curves of degradable epoxy resin under different heating rates are shown in Figure 4. It can be observed that resins at different heating rates exhibit distinct and single exothermic peaks, indicating that the pre-curing reaction of the resin is completed in one step. There is a clear correlation between the exothermic peak of resin and the heating rate. As the heating rate increases, the exothermic peak shifts towards a high temperature direction and the amplitude of the peak increases. This is because the heat flux rate is directly proportional to the heating rate. As the heating rate increases, the thermal effect generated per unit time increases accordingly; the thermal inertia and temperature difference also increase [27]. Therefore, the exothermic peak moves towards a high temperature. In the DSC curve, the temperature *T*_i_ at the beginning of the curing reaction, the peak temperature *T*_p_ of the curing reaction, and the temperature *T*_f_ at the end of the curing reaction, can also be obtained, all of which move towards the high-temperature zone with the increase in the heating rate, as shown in Table 2.

The value of *E*_a_ and *A* can be obtained from the Kissinger equation [28,29,30]:

ln (*β*/*T*^2^_p_) = ln [(*AR*/*E*_a_) − *E*_a_/(*RT*_p_)] (3)

**Figure 5:**
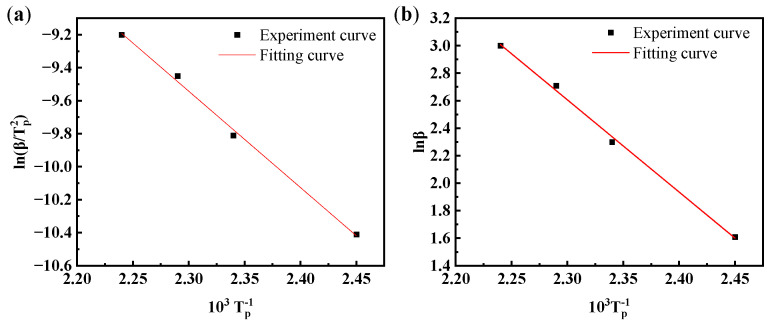
Fitting curve of degradable epoxy resin curing kinetics: (**a**) appearance activation energy and pre-exponential factor, (**b**) reaction order.

According to Equation (3), ln (*β*/*T*^2^_p_) draws a graph of 1/*T*_p_ and performs linear fitting. The activation energy *E*_a_ of the curing reaction can be calculated by the slope, and the pre-exponential factor *A* can be obtained by the intercept. The Kissinger equation fitting curve is shown in Figure 5a; a good linear correlation was obtained with a slope of −5.84. Substituting the obtained slope and intercept, it can be obtained that *E*_a_ = 48.55 KJ/mol, *A* = 2.85 × 10^5^ min^−1^.

The reaction order *n* can be obtained from the Crane equation [31]:

d(ln*β*)/d(1/*T*_p_) ≈ −*E*_a_/*nR* (4)

The corresponding Crane equation fitting curve is exhibited in Figure 5b. A good linear correlation is also obtained with a slope and an intercept of −6.70 and 18.02, respectively. Substituting the obtained slope and intercept into Equation (4), *n* is calculated to be 0.87. If we substitute the apparent activation energy, pre-exponential factor, and reaction order into the curing reaction kinetics formula (Equation (2)), the curing reaction kinetics formula can be obtained.

**Figure 6:**
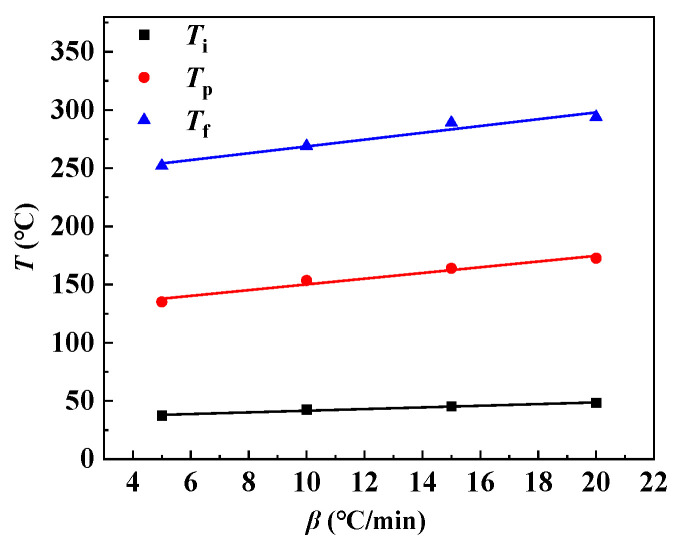
Extrapolation curves of the curing temperature.

In addition, the corresponding extrapolation curve about the curing temperature of RFRDE is shown in Figure 6. The correlation coefficients of the three fitted curves (*T*_i_, *T*_p_, *T*_f_) are 0.96, 0.95, and 0.93, respectively, indicating a good linear relationship with the fitted curves. The extrapolated curing temperatures (*T*_i_* (theoretical gel temperature), *T*_p_* (theoretical curing temperature), *T*_f_* (theoretical post-treatment temperature)) for RFRDE were obtained, which were 34.52 °C, 125.55 °C, and 239.50 °C, respectively. Therefore, it can be heated from 34.52 °C to 125.55 °C at a certain heating rate, kept for a period of time, and then raised to 239.50 °C for post-treatment to allow the resin to fully react.

**Figure 7:**
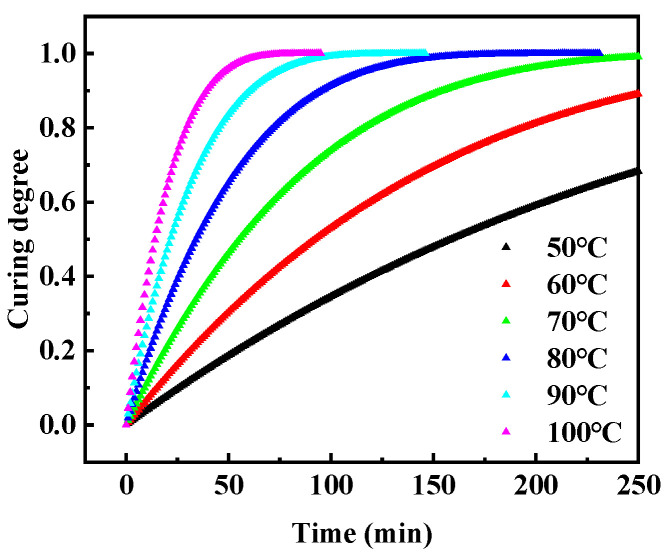
Curves of the ideal curing degree–temperature–time of the degradable epoxy resin.

Furthermore, the ideal curing degree of RFRDE under different temperature and constant temperature conditions can be extracted from Equation (2). As shown in Figure 7, It can be seen that the curing rate of RFRDE increases as the temperature increases. Under the same temperature conditions, the curing degree of RFRDE increases with time. As the temperature increases, the time required for complete solidification shortens. When the temperature reaches 100 °C, it only takes 75 min as the solidification degree reaches 1.

Due to the significant influence of the heat treatment temperature on the tensile performance of ramie fibers, Table 3 lists four types of specimens with the highest curing temperatures of 100 °C, 125 °C, 150 °C, and 180 °C, respectively. From the table, it can be seen that when the highest curing temperature is 100 °C, the tensile strength of RFRDE reaches the highest value. This value is higher than the average values recorded for the composites reinforced with carbon fibers or inorganic fibers (Table S1). As the highest curing temperature increases, the tensile strength of the composite material decreases. On the contrary, the tensile modulus and curing degree increase. During the winding process of fibers, resin enters the interior of the fibers, reducing the impact of moisture on fiber properties. When the temperature is higher than the initial curing temperature, resin molecules begin to crosslink, and the reaction is most intense when the temperature reaches the highest curing temperature [32]. When the curing termination temperature is reached, the effect of prolonging curing on improving the curing degree is negligible. Usually, a two-stage system (*T*_1_, *T*_2_) is used for resin curing. *T*_1_ is generally chosen to be greater than the initial curing temperature but lower than the maximum curing temperature to prevent a decrease in the curing degree caused by excessive reaction in the first stage. *T*_2_ is generally higher than or equal to the maximum curing temperature to achieve the goal of improving the resin curing degree. Considering the influence of the heat treatment temperature on the mechanical properties of ramie fibers, this study chose a curing system of 60 °C/2 h–100 °C/5 h; furthermore, the curing degree can be improved by appropriately extending the curing time of the resin.

### 3.3. The Effect of Different Treatment Processes on the Interface Strength between Polyethylene Plastics and RFRDE

**Figure 8:**
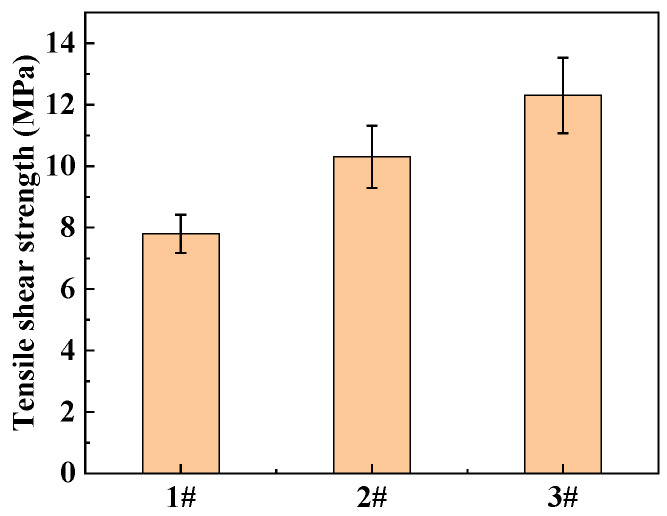
Different treatment processing-tensile shear strength, 1# Corona, 2# Adhesive, 3# Corona, and adhesive. The error bars represent the standard deviations of five representative measurements.

Due to its low surface energy and the absence of polar groups on the surface, the surface of polyethylene is not active. In addition, it has a highly symmetrical methylene structure, resulting in poor adhesion between it and the fiber layer. A low interface strength is not conducive to wrapping of type IV gas cylinders. Figure 8 and Table S2 show shear strengths after three different surface treatment processes (corona, adhesive, corona and adhesive). As shown in Figure 8, the third treatment method can yield maximum adhesive strength at the interface. The corona treatment method can generate a certain etching effect on the surface of polyethylene, increase its surface roughness, and increase the contact area with the matrix, resulting in a decrease in the contact angle and an improvement in the wettability between fibers and the resin [33,34,35]. At the same time, this method can also generate a large number of polar functional groups on the surface of the material, form chemical bonds with the resin, and improve the interlayer shear strength between them [36].

**Figure 9:**
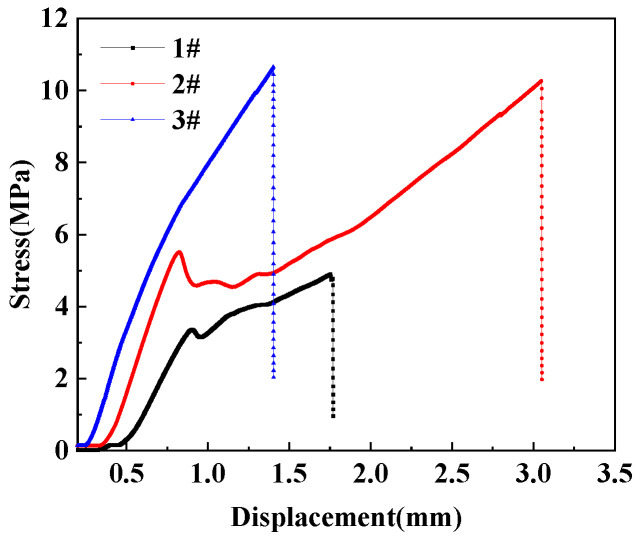
Different treatment processing-tensile shear strength, 1# Corona, 2# Adhesive, 3# Corona, and adhesive.

**Figure 10:**
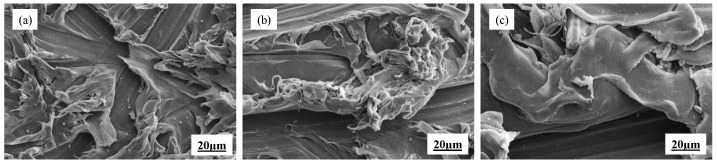
SEM images of the tensile shear interface: (**a**) corona treatment, (**b**) adhesive treatment, (**c**) corona and adhesive treatment.

To analysis the relativity of displacement and tensile shear stress, corresponding curves of different treatment processing of PE/RFRDE are shown in Figure 9. Due to the use of flexible adhesives, the stiffness matching adaptability of the PE and RFRDE bonding interfaces is enhanced when subjected to tensile and shear forces, resulting in an improvement in the adhesive’s bonding ability. After corona discharge and adhesive treatment, the connection strength did not change and the modulus in the early stage was consistent with that of the adhesive. However, in the later stage of deformation, the fracture extension performance was improved, which enhances the adhesive performance. Further analysis of the microstructure of the adhesive interface and fracture morphology was observed in Figure 10. After the tensile shear test, the amount of adhesive driven by different treatment methods on the surface of polyethylene varies. From the comparison of Figure 10a–c, it can be seen that the surface in Figure 10c is relatively rougher, and the integration of the resin and fibers is higher. Due to the increased concavity and convexity of the polyethylene surface, the adhesive strength is improved. Furthermore, the corona treatment significantly improves the wetting performance of epoxy resin on PE. In addition, when adhesives with better fluidity and wettability are used, the interface resin disperses better on the lining surface, resulting in a significant increase in the interface strength between the composite material and the lining, thereby enhancing the reliability and strength of the adhesion.

### 3.4. The Influence of Winding Tension on the Burst Strength of Gas Cylinders

**Figure 11:**
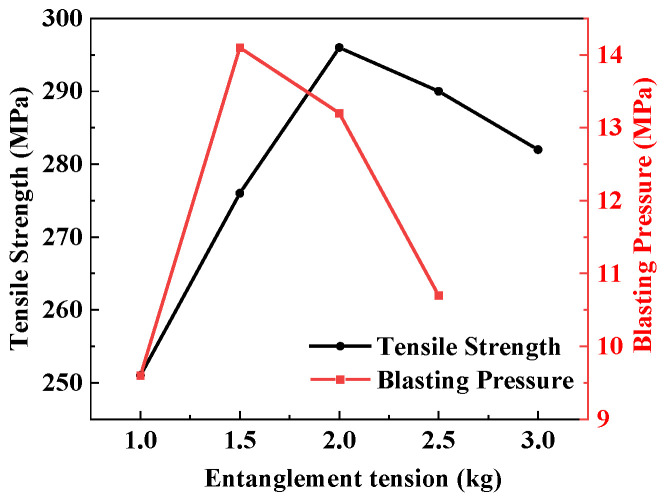
Tensile strength and blasting pressure curves against entanglement tension.

Different winding tensions have a significant impact on the burst strength of composite gas cylinders. To obtain the optimal winding tension, Figure 11 shows the tensile strength and blasting pressure of composite gas cylinders under six different entanglement tensions. The fiber tensile strength reaches its maximum at a winding tension of 2 kg, but the burst pressure reaches its peak at 1.5 kg. As the winding tension increases, the friction between the fiber and the guide roller increases, and the damage to the fiber also increases. During the winding process, fiber wear is an important factor affecting the strength conversion of the fiber. The increase in winding tension can, on the one hand, increase the volume fraction of fibers in composite materials and the conversion rate of mechanical properties of fibers in composite materials, thereby improving the mechanical properties of composite materials. On the other hand, the degree of fiber damage also increases with the increase in the winding tension, leading to a decrease in the mechanical properties of the wound fibers, thereby reducing the mechanical properties of the composite material.

**Figure 12:**
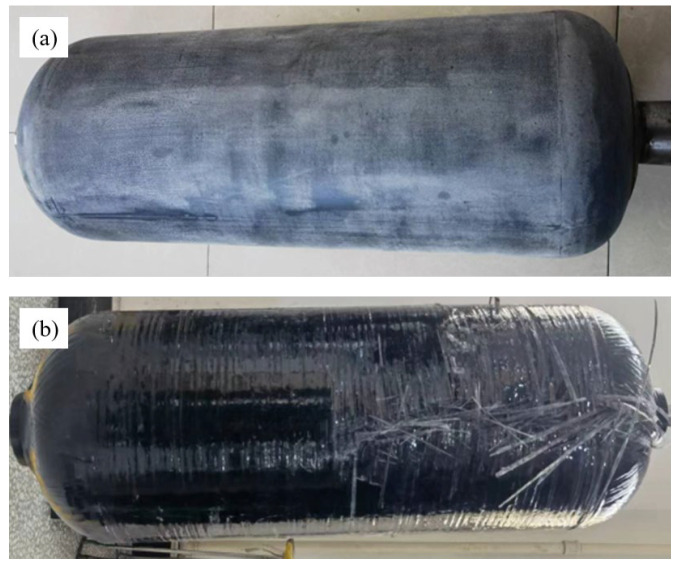
(**a**) Surface of PE lining treated with corona discharge and adhesive treatment. (**b**) Picture of gas cylinder after blasting with 1.5 kg winding tension.

Selecting a winding tension of 1.5 kg for gas cylinder molding, Figure 12a shows the corona treatment before winding the gas cylinder and Figure 12b shows the damage to the gas cylinder after blasting. It can be seen that the aging form of the gas cylinder is mainly a fiber fracture and delamination. This phenomenon indicates that the interface effect between the ramie fiber and resin, as well as the interface effect between RFRDE and liner, will directly affect the explosive performance of gas cylinders.

## 4. Conclusions

This study analyzed and characterized the thermodynamic properties of RFRDE and optimized the interface bonding process between polyethylene plastic and RFRDE. At the same time, by adjusting the winding tension of ramie fibers, the IV type gas cylinder reached the optimal bursting pressure. The main conclusions are as follows:

(1) The tensile strength of ramie fiber bundles decreases with an increasing temperature, and when the heat treatment temperature reaches 180 °C the tensile strength decreases by 34% compared to untreated fibers. The thermal weight loss test was conducted on ramie fibers, and the fiber showed the most significant thermal weight loss effect within the range of 300–400 °C. The residual weight of ramie fibers heated to 500 °C in a N_2_ atmosphere is 20%, and compared to the air environment (0%), the thermal weight loss of ramie fibers in a nitrogen environment is less.

(2) Through DSC testing and curing kinetics calculations, the *T*_i_*, *T*_p_*, and *T*_f_* of degradable epoxy resin were 34.52 °C, 125.55 °C, and 239.50 °C, respectively. At the same time, mechanical tests on RFRDE under different highest curing temperature conditions showed that the tensile strength of RFRDE decreased by about 19.93% at 180 °C compared to the highest curing temperature of 100 °C. The highest value (cured at 100 °C) is 30% higher than the average values recorded for the composites reinforced with carbon fibers or inorganic fibers. Furthermore, as the highest curing temperature increases, the curing degree of the composite material also increases accordingly.

(3) The use of corona discharge and adhesive can effectively improve the bonding strength between polyethylene plastic and RFRDE, thereby enhancing the interface performance between polyethylene lining and fibers. In addition, through NOL ring testing on ramie fibers, it was found that when the winding tension was 1.5 kg, the maximum burst pressure of a type IV gas cylinder was 14.1 MPa. The gas cylinder after a burst mainly exhibited fiber cracking and delamination, indicating that improving the RFRDE interface strength has a significant effect on optimizing the gas cylinder burst.

## Figures and Tables

**Table 1:** Bundled yarn strength and decline in percentage of different heat treatment temperatures on ramie fiber.

	Untreated	100 °C	125 °C	150 °C	180 °C
Bundled yarn strength (MPa)	710	575	553	518	469
Decline in percentage (%)	/ *	19	22	29	34

* as it is the control group, the untreated sample has no decrease rate.

**Table 2:** Thermodynamic parameters at different heating rates.

Heating Rate (*β*, °C/min)	*T*_i_ (°C)	*T*_p_ (°C)	*T*_f_ (°C)	Δ*H* (J/g)
5	37.4 ± 0.1	135.1 ± 0.7	252.1 ± 0.8	−374.9 ± 0.5
10	42.5 ± 0.3	153.5 ± 0.4	268.9 ± 0.3	−373.2 ± 0.2
15	45.5 ± 0.6	163.9 ± 0.2	289.3 ± 0.6	−347.2 ± 0.3
20	48.3 ± 0.2	172.6 ± 0.1	294.2 ± 0.2	−377.8 ± 0.1

**Table 3:** Tensile strength, modulus, and curing degree of RFRDE at different curing temperatures.

Curing Temperature	Tensile Strength (MPa)	Tensile Modulus (GPa)	Curing Degree (%)
100 °C	296 ± 12	21 ± 0.8	85 ± 4
125 °C	288 ± 9	25 ± 0.5	91 ± 3
150 °C	281 ± 13	25.6 ± 0.3	95 ± 1
180 °C	237 ± 7	26.3 ± 0.7	98 ± 2

## Data Availability

Data are contained within the article.

## Supplementary Materials

The following supporting information can be downloaded at: https://www.mdpi.com/article/10.3390/ma17194802/s1, Table S1: Mechanical properties of fiber-based epoxy resin; Table S2: Tensile shear strength of different treatment processing.
