# Supplementary material for: Thermomechanical Properties of Ramie Fiber/Degradable Epoxy Resin Composites and Their Performance on Cylinder Inner Lining
Source: Materials (Basel). 2024 Sep 29;17(19):4802. doi: 10.3390/ma17194802 (PMC11478300; doi:10.3390/ma17194802)
Supplement: Supplementary file 1 [file materials-17-04802-s001.zip › materials-3145355-supplementary.pdf]

Table S1. Mechanical properties of fiber-based epoxy resin.

Samples	Tensile Strength (MPa)	Tensile Modules (GPa)	References
Neat epoxy	73	2.5	J. Compos. Sci. 2021, 5, 89
Carbon fiber/epoxy	120	9.1	
Basalt fiber/epoxy	125	7.2	
Aramid fiber/epoxy	362	12.4	

Table S2. Tensile shear strength of different treatment processing.

Sample Number	Treatment Processing	Tensile Shear Strength (MPa)
#1	Corona	7.8
#2	Adhesive	10.3
#3	Corona and Adhesive	12.4
